# Targeting mGlu5 Metabotropic Glutamate Receptors in the Treatment of Cognitive Dysfunction in a Mouse Model of Phenylketonuria

**DOI:** 10.3389/fnins.2018.00154

**Published:** 2018-03-16

**Authors:** Francesca Nardecchia, Rosamaria Orlando, Luisa Iacovelli, Marco Colamartino, Elena Fiori, Vincenzo Leuzzi, Sonia Piccinin, Robert Nistico, Stefano Puglisi-Allegra, Luisa Di Menna, Giuseppe Battaglia, Ferdinando Nicoletti, Tiziana Pascucci

**Affiliations:** ^1^Department of Physiology and Pharmacology, Sapienza Università di Roma, Rome, Italy; ^2^Department of Pediatrics and Child Neuropsychiatry, Sapienza Università di Roma, Rome, Italy; ^3^Daniel Bovet Department of Psychology, Neurobiology Research Center, Sapienza Università di Roma, Rome, Italy; ^4^Department of Biology, Università degli Studi di Roma Tor Vergata, Rome, Italy; ^5^IRCCS Foundation Santa Lucia, Rome, Italy; ^6^IRCCS Neuromed, Pozzilli, Italy

**Keywords:** phenylketonuria, mGluR5, hippocampus, intellectual disability, ENU2 mice

## Abstract

We studied group-I metabotropic glutamate (mGlu) receptors in Pah^enu2^ (ENU2) mice, which mimic the genetics and neurobiology of human phenylketonuria (PKU), a metabolic disorder characterized, if untreated, by autism, and intellectual disability (ID). Male ENU2 mice showed increased mGlu5 receptor protein levels in the hippocampus and corpus striatum (but not in the prefrontal cortex) whereas the transcript of the mGlu5 receptor was unchanged. No changes in mGlu1 receptor mRNA and protein levels were found in any of the three brain regions of ENU2 mice. We extended the analysis to Homer proteins, which act as scaffolds by linking mGlu1 and mGlu5 receptors to effector proteins. Expression of the long isoforms of Homer was significantly reduced in the hippocampus of ENU2 mice, whereas levels of the short Homer isoform (Homer 1a) were unchanged. mGlu5 receptors were less associated to immunoprecipitated Homer in the hippocampus of ENU2 mice. The lack of mGlu5 receptor-mediated long-term depression (LTD) in wild-type mice (of BTBR strain) precluded the analysis of hippocampal synaptic plasticity in ENU2 mice. We therefore performed a behavioral analysis to examine whether pharmacological blockade of mGlu5 receptors could correct behavioral abnormalities in ENU2 mice. Using the same apparatus we sequentially assessed locomotor activity, object exploration, and spatial object recognition (spatial novelty test) after displacing some of the objects from their original position in the arena. Systemic treatment with the mGlu5 receptor antagonist, MPEP (20 mg/kg, i.p.), had a striking effect in the spatial novelty test by substantially increasing the time spent in exploring the displaced objects in ENU2 mice (but not in wild-type mice). These suggest a role for mGlu5 receptors in the pathophysiology of ID in PKU and suggest that, also in adult untreated animals, cognitive dysfunction may be improved by targeting these receptors with an appropriate therapy.

## Introduction

Phenylketonuria (PKU), one of the most common inherited inborn errors of metabolism, is caused by the deficiency of the enzyme phenylalanine hydroxylase (PAH), which catalyzes the conversion of the phenylalanine (Phe) into tyrosine. Clinical manifestations of PKU are largely due to the accumulation of Phe in the CNS. If untreated, children affected by PKU show intellectual disability (ID) associated with autism, seizures, and motor deficits (Blau et al., 2010). The introduction of newborn screening programs and early treatment with a low-Phe diet has considerably improved the CNS manifestations of PKU. However, the clinical outcome of PKU is suboptimal, and early-treated patients still exhibit lower intelligence quotient (IQ) and additional neuropsychiatric problems (Smith et al., 1990; DeRoche and Welsh, 2008; Burton et al., 2013; Nardecchia et al., 2015; Manti et al., 2016). This stimulates the search for new drug targets for the treatment of CNS manifestations associated with PKU.

Although PKU has been studied for decades, the pathophysiology of ID remains unclear. Increased Phe concentrations disrupt neurotransmitter metabolism, white matter integrity, and synapse functioning in PKU patients and in experimental animal models of PKU (Dyer et al., 1996; Puglisi-Allegra et al., 2000; Martynyuk et al., 2005). BTBR-Pah^enu2^ mice (ENU2 mice), derived from chemically induced mutation of the PAH gene in male BTBR mice, closely mimic the genetics, biochemistry, and neurobiology of PKU in humans (Shedlovsky et al., 1993; McDonald and Charlton, 1997). These mice show defects in simple discrimination learning, short-term memory, reference memory, habit learning, and spatial and visual memory (Sarkissian et al., 2000; Cabib et al., 2003; Pascucci et al., 2013), associated with a reduced density of dendritic spines, shortened length of the presynaptic active zone, widened synaptic cleft, decreased thickness of postsynaptic density, reduced percentage of mature spines, and decreased levels of proteins associated with synaptic functioning and alterations in excitatory/inhibitory ratio in cortical circuitry (Hörster et al., 2006; Andolina et al., 2011; Liang et al., 2011; Horling et al., 2015; De Jaco et al., 2017). The neurochemical abnormalities underlying these behavioral and morphological changes are only partially elucidated. ENU2 mice show large reductions in brain serotonin levels, which result from Phe-induced inhibition of tryptophan hydroxylase, and a lesser reduction in catecholamine level (Puglisi-Allegra et al., 2000; Pascucci et al., 2009; Andolina et al., 2011). Abnormalities of glutamatergic neurotransmission have been found to be associated with PKU. Accordingly, Phe, at concentrations similar to those found in PKU, was reported to inhibit NMDA receptors function, and to depress the amplitude and frequency of excitatory postsynaptic currents in cultured hippocampal neurons (Glushakov et al., 2002, 2003). In addition, increases in the density of MK-801 recognition site on the NMDA gated ion channel, and changes in the expression of NMDA and AMPA receptor subunits (increases in GluN2A, GluA1, and GluA2/3 and reductions of GluN2B) were reported in the forebrain of PKU mice (Glushakov et al., 2005; Martynyuk et al., 2005).

To our knowledge, no studies have been performed on metabotropic glutamate (mGlu) receptors in PKU models, although these receptors are involved in mechanisms of activity-dependent synaptic plasticity underlying learning and memory processes (Nicoletti et al., 2011). In particular, abnormalities in group-I mGlu receptors (mGlu1 and mGlu5 receptors) have been linked to cognitive dysfunction in models of disorders characterized by autism and ID, such as Fragile-X, Rett's syndrome, Angelmann's syndrome, tuberous sclerosis, and chromosome 16p11.2 microdeletion (Huber et al., 2002; Auerbach et al., 2011; Michalon et al., 2012; D'Antoni et al., 2014; Pignatelli et al., 2014; Tian et al., 2015). Group-I mGlu receptors interact with Homer proteins, which physically link the C-terminal domain of mGlu1α and mGlu5 receptors to scaffolding and effector proteins and ion channels in the postsynaptic densities, such as the inositol-1,4,5-trisphosphate receptor and TrpC channels (Brakeman et al., 1997; Tu et al., 1998). Two types of Homer proteins have been identified: (i) long Homer isoforms, which include Homer 1b,−1c,−2, and −3 and may interact to each other by means of the coiled-coil domain in their C-terminal portion; and (ii) the shorter Homer 1a isoform, which is unable to interact with other Homer proteins and is induced by synaptic hyperactivity (Brakeman et al., 1997; Xiao et al., 1998, 2000; Fagni et al., 2000). Abnormalities in the interaction between mGlu5 receptors and Homer have been described in animal models of monogenic autism (Ronesi et al., 2012; D'Antoni et al., 2014; Pignatelli et al., 2014; Guo et al., 2015, 2016), suggesting that Homer expression and function lies at the core of postsynaptic mechanisms underlying learning and memory processes and cognitive functions. We now report that expression of mGlu5 receptors and long Homer isoforms is altered in the brain of ENU2 mice, and that pharmacological blockade of mGlu5 receptors is able to reverse behavioral abnormalities in these mice.

## Materials and methods

### Animals

Eighty days old homozygous (−/−) Pah^Enu2^ (ENU2) and (+/+) Pah^Enu2^ (WT) male mice of BTBR background strain were used for all experiments and were obtained by heterozygous mating. In a separate set of experiments, we used 3 male ENU2, 4 female ENU2 and 7 female heterozygous (+/–; HTZ) mice for the study of a possible gender effect (age: 40–60 days).

Genetic characterization was performed on DNA prepared from tail tissue using the Easy DNA kit (Invitrogen, Carlsbad, CA, USA). The ethylnitrosourea (enu2) mutation was detected after PCR amplification of exon 7 of the *Pah* gene and digestion thought restriction enzyme BsmAI (NewEnglandBiolabs, Inc., USA) as previously described (Pascucci et al., 2008). Mice were weaned at postnatal day (PND) 21, experimental subjects (sex matched) from different litters were housed 2–4 per cage with food and water *ad libitum* on a 12:12 h dark: light cycle (light on 07.00 a.m.–07.00 p.m. h).

All efforts were made to minimize the number of animals used and to alleviate their discomfort. All experimental procedures were performed in strict compliance with the Italian (D.L. 26/2014) and European Union Directive (2010/63/EU) on the protection of animals used for scientific purposes. All animal experiments were approved by the Italian Ministry of Health (Rome, Italy).

Brain tissue was collected from ENU2 and WT mice.

### Drug and treatment

2-methyl-6-(phenylethynyl)-pyridine hydrochloride (MPEP, Tocris Bioscience) was dissolved in saline and injected i.p. at the dose of 20 mg/kg, 30 min before the behavioral testing session. This dose of MPEP was found to be behaviorally effective in BTBR mice (Silverman et al., 2010).

### Immunoblotting

Mice were decapitated and the prefrontal cortex, hippocampus, and corpus striatum were removed. The medial portion of the frontal lobe (anterior to the head of the caudate nucleus) containing the anterior cingulate cortex, prelimbic cortex, and infralimbic cortex was carefully dissected by hand and considered as “prefrontal cortex.” Tissues were stored at −80°C. Tissues were homogenized and proteins extracted as previously described (Orlando et al., 2014). Proteins (15–25 μg) were resuspended in SDS-bromophenol blue reducing buffer containing 5% 2-mercaptoethanol and separated by electrophoresis on 8% (or 10% for Homer) SDS polyacrylamide gels. Samples were never boiled (for mGlu receptors), or were incubated at 65°C for 5 min (for Homer), before loading. The proteins were transferred onto nitrocellulose membranes (350 mA, 1 h, 4°C) and thereafter, membranes were blocked for unspecific binding with 5% non-fat dry milk in TBST (TBS containing 0.1% Tween 20) for 1 h at RT. Membranes were incubated with the following primary antibodies: mouse anti-mGlu1α (DB Bioscience, 556389), 1:1,000, overnight at 4°C; rabbit anti-mGluR5 (Millipore, cat AB5675), 1:5,000, 1 h at RT; mouse anti-β-actin (Sigma-Aldrich, Milan, Italy, cat. A5316), 1:10,000, 1 h at RT; mouse anti-Homer (Santa Cruz, sc-17842), 1:1,000, 1 h at RT; goat anti-Homer 1a (Santa Cruz, sc-8922), 1:2,000, 1.5 h at RT. Immunoreactive bands were visualized by enhanced chemiluminescence (GE Healthcare, Milan, Italy) using horseradish peroxidase-conjugated secondary antibodies (Cell Signaling Technology, Danvers, MA).

### Co-immunoprecipitation

Hippocampi were homogenized at 4°C in co-immunoprecipitation buffer (50 mM Tris, pH 7.4, 120 mM NaCl, 0.5% Igepal, 1 mM EDTA, 1 mM EGTA), and 1 mg of total proteins were tumbled overnight at 4°C with 5 μg of anti pan-Homer antibodies (Santa Cruz Biotechnology, D-3). Protein A/G agarose bead slurry (Thermo Fisher Scientific) was added for two additional hours, and the beads were then washed with co-immunoprecipitation buffer. Western blotting was performed with anti-mGlu5 and anti pan-Homer antibodies.

### RNA isolation and RT-PCR

Total RNA was extracted from tissues (cortex, hippocampus, and corpus striatum) with TRI reagent (Sigma Aldrich, Milan, Italy) according to manufacturer's instructions, and quantified by spectrophotometric analysis. RNA samples were digested with DNAse (Promega, Madison, WI) and single strand cDNA was synthesized from 1 μg of total RNA using Superscript II (Promega, Madison, WI) and random hexamers. Real-time PCR was performed on 20 ng of cDNA by using specific primers and SYBR Green Master Mix (Bioline, London, UK) on an iCycler Biorad instrument (Hercules, CA). Thermal cycler conditions were as follows: 10 min at 95°C (polymerase activation) followed by 40 cycles of denaturation at 95°C for 15 s, annealing at 58°C for 15 s, extension at 72°C for 15 s.

Sequences of primers used were as follows: Homer 1a (NM_011982): for 5′-TCTTCAGTC TCCTTTGACACCA-3′ and rev 5′-CATGATTGCTGAATTGAATGTG-3′; pan-Homer 1 (NM_001284189): for 5′-TGGACTGGGATTCTCCTCTG-3′ and rev 5′-TGTGTCACATCGGGTGTTCT-3′; mGlu5 receptor (NM_001081414): for 5′-ACGAAGACCAACCGTATTGC-3′ and rev 5′-AGACTTCTCGGATGCTTGGA-3′; mGlu1 receptor (NM_016976): for 5′-CATACGGAAAGGGGAAGTGA-3′ and rev 5′-AAAAGGCGATGGCTATGATG-3′; b-actin (NM_007393): For 5′-GTTGACATCCGTAAAGACC-3′ and rev 5′-TGGAAGGTGGACAGTGAG-3′.

mRNA quantities were calculated from serially diluted standard curves simultaneously amplified with the samples and normalized vs. β-actin mRNA levels.

### Electrophysiology

Brains were rapidly dissected out and slices from hippocampus (250–400 μm) were cut in ice-cold artificial cerebrospinal fluid (ACSF) composed of (in mM): NaCl 124, KCl 3.0, MgCl_2_ 1.0, CaCl_2_ 2.0, NaH_2_PO_4_ 1.25, NaHCO_3_ 26, glucose 10, saturated with 95% O_2_, 5% CO_2_, pH 7.4. The CA3 region was not removed from the slices. Slices were allowed to recover for 2–4 h and then placed on a nylon mesh, completely submerged in a small chamber (0.8 ml), and superfused with oxygenated ACSF (30–31°C) at a constant flow rate of 2.5–3.0 ml/min. The slope of the field EPSPs (fEPSPs) was recorded from the apical dendrite layer of the CA1 pyramidal cells by means of saline-filled glass electrodes of ~2–4 MΩ resistance. Stimulating monopolar electrodes were placed in Schaffer collateral/commissural afferents, and stimulation amplitude was adjusted so as to produce one-half of the maximal response. Signals were filtered at 3 kHz and digitized at 10 kHz. After the stabilization of the fEPSP, LTD was induced by low-frequency stimulation (1 Hz for 15 min) or following (S)-3,5-dihydroxyphenylglycine [(S)-DHPG] application (50 μM, 10 min).

### Behavioral analyses

#### Spatial novelty test

The apparatus was a circular open field (60 cm in diameter and 20 cm in height). The apparatus floor was gray, divided into sectors using black lines, and covered by a transparent plastic lining. It was surrounded by a gray plastic wall rendering the visual environment homogeneous, except for a black and white striped pattern (30 × 20 cm) that was attached to the wall of the field as local cue. Mice were individually submitted to five successive videotaped sessions, each 6-min long, with 3-min interval. At the end of the last session mice were placed back in their home-cages. Session 1 (S1) was used to habituate the animal to the apparatus. Distance moved and velocity were assessed as locomotor activity in an open-field condition. In the sessions 2, 3, and 4 (S2, S3, and S4), each mouse was introduced in the same sector of the open field where four different objects (called A, B, C, and D) were placed in the same position. During these sessions mice could learn the spatial configuration of the objects included in the apparatus. Object exploration was expressed by the time (s) spent in contact with the objects (contact: the snout of the animal touching an object).

In last test session (S5) two of the four objects were moved from the original position (object A was moved in the position previously taken by object B and the latter was placed in a new location) to test the animal's reaction to a spatial change (discrimination of spatial novelty). In this session, the exploration of two categories of objects, displaced object (DO) and non-displaced object (NDO), were collected. These measures were expressed as the mean time in contact with the objects belonging to the two categories in each test session minus the mean time spent in contact with the same object category in the previous session (S5–S4). Apparatus and objects were cleaned between each mouse tested (Cabib et al., 2003).

#### Statistical analysis

Data were analyzed using Student's *t*-test, two-way or three-way analysis of variance (ANOVA). Fisher's protected least-significant difference (PLSD) test was used for comparing group means only when a significant *F*-value was determined by ANOVA. Values are expressed as the mean ± SEM. Significance was set at *p* < 0.05.

## Results

### Up-regulation of mGlu5 receptors in the hippocampus and corpus striatum of ENU2 mice

**Figure 1:**
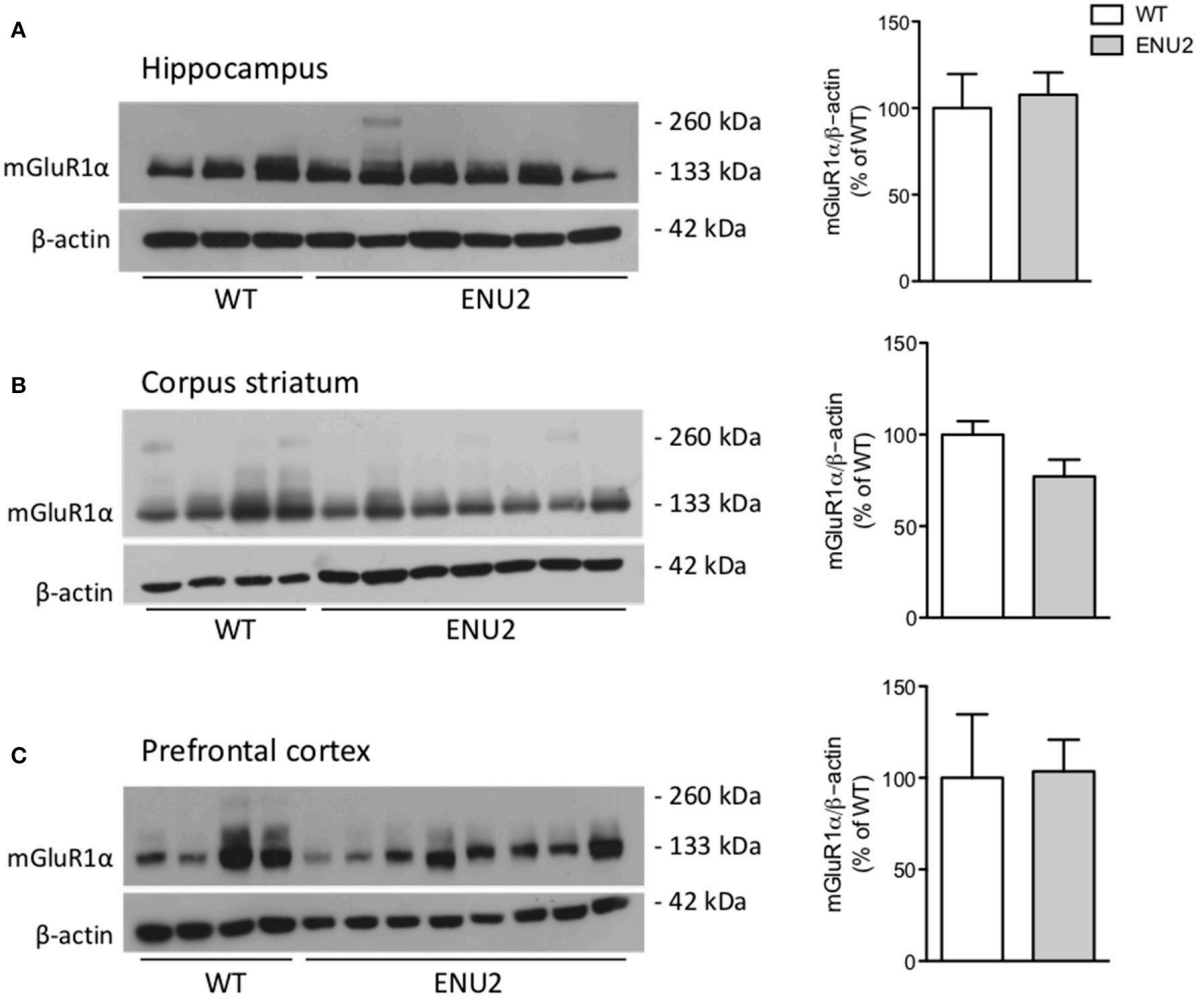
No changes in mGlu1α receptor protein levels in the hippocampus **(A)**, corpus striatum **(B)**, and prefrontal cortex **(C)** of ENU2 mice. All samples from the hippocampus and prefrontal cortex of ENU2 and WT mice are shown in the immunoblots in **(A,C)**, respectively. A representative immunoblot of striatal samples is shown in **(B)**. Values are means ± S.E.M. of 3 WT and 6 ENU2 mice in **(A)**; 10 WT and 14 ENU2 mice in **(B)**; and 4 WT and 8 ENU2 mice in **(C)**.

We examined whether the PKU phenotype was associated with changes in the expression of mGlu1α and mGlu5 receptors in brain regions that play key roles in cognitive function and motor programming (hippocampus, corpus striatum, and prefrontal cortex). Western blot analysis of mGlu1α receptors showed a band at about 130–135 KDa corresponding to receptor monomers. No changes in mGlu1α receptor protein levels were found in any brain region of ENU2 mice as compared to their WT counterparts (Figure 1).

**Figure 2:**
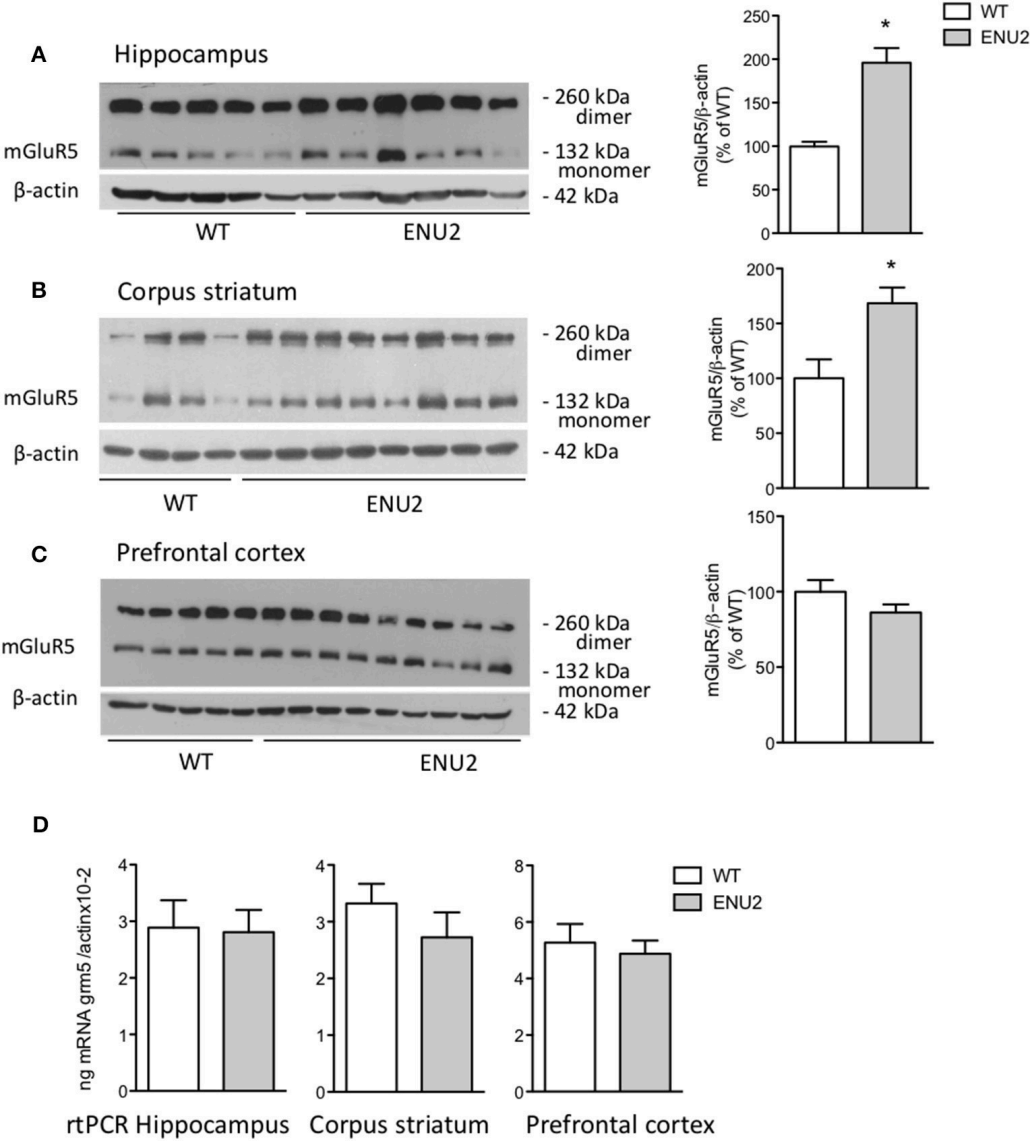
Increased mGlu5 receptor levels in the hippocampus and corpus striatum of ENU2 mice. Representative immunoblots of samples from the hippocampus, corpus striatum, and prefrontal cortex are shown in **(A–C)**, respectively. Densitometric values are means ± S.E.M. of 8 WT and 12 ENU2 mice in **(A)**; 10 WT and 14 ENU2 mice in **(B)**; and 9 WT and 17 ENU2 mice in **(C)**. ^*^p < 0.05 vs. the respective WT-values [Student's *t*-test; *t*_(18)_ = 4.51 in **(A)**; *t*_(22)_ = 3.06 in **(B)**; *t*_(24)_ = 1.49 in **(C)]**. mGlu5 mRNA levels in the hippocampus, corpus striatum, and prefrontal cortex are shown in **(D)**, were values are means ± S.E.M. of 5 WT and 4 ENU2 mice for the hippocampus; 5 WT and 5 ENU2 mice for the corpus striatum; and 4 WT and 6 ENU2 mice for the prefrontal cortex.

Immunoblots with mGlu5 receptor antibodies showed a 130 kDa band corresponding to receptor monomers, and a second band at the expected molecular size (250 kDa) of receptor dimers (Figures 2A–C). Densitometric analysis was performed as the sums of the dimer and monomer densitometry values. Interestingly, mGlu5 receptor protein levels were markedly enhanced in the hippocampus and striatum of ENU2 mice, with no changes in the prefrontal cortex (Figures 2A–C). In contrast, mGlu5 receptor mRNA levels were unchanged in the three brain regions of ENU2 mice (Figure 2D).

**Figure 3:**
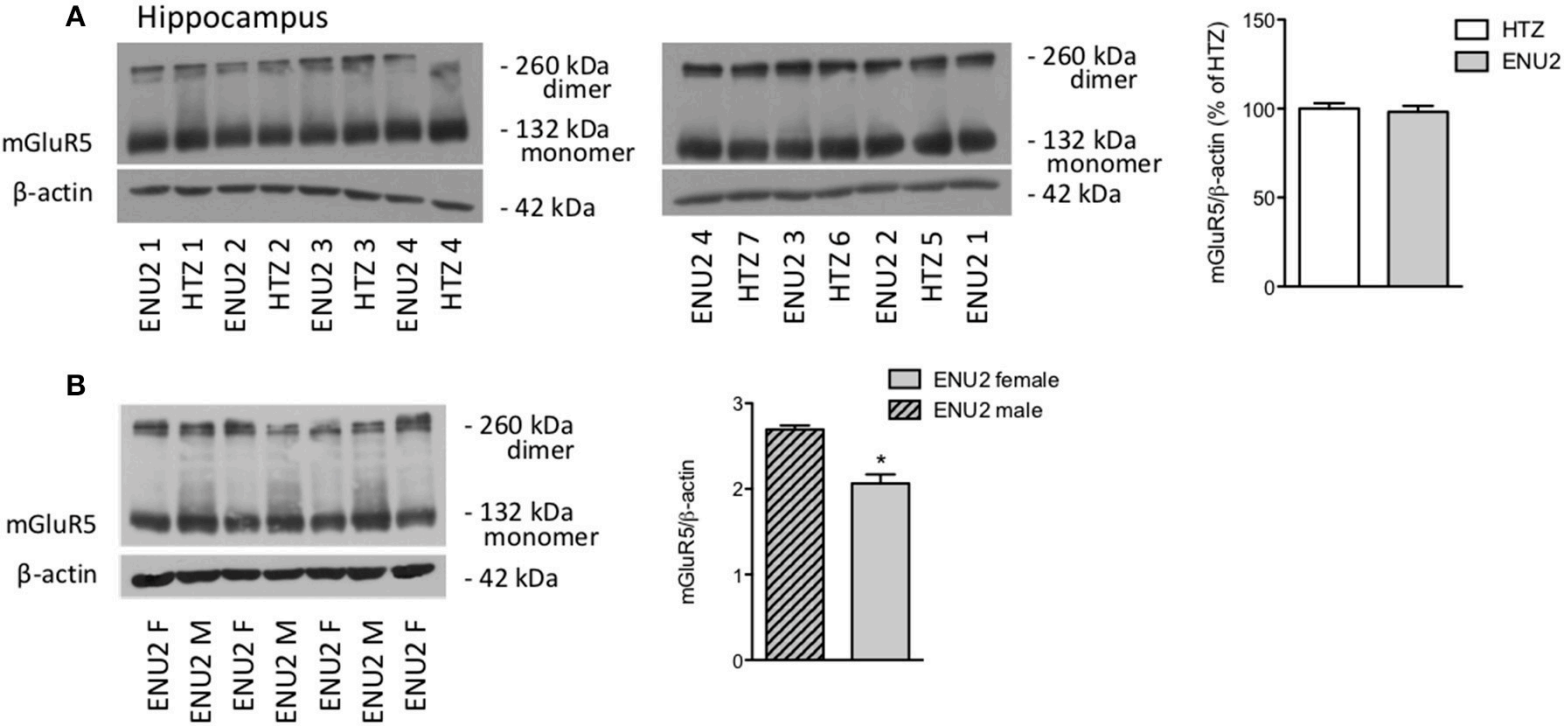
The increase in mGlu5 receptor protein levels found in ENU2 mice is gender-dependent. Immunoblot analysis of mGlu5 receptors in the hippocampus of female ENU2 (*n* = 4) and heterozygous (HTZ, *n* = 7) mice is shown in **(A)**. Comparison between male (*n* = 3) and female (*n* = 4) ENU2 mice is shown in **(B)**. Densitometric values are means ± S.E.M. In **(B)**, ^*^p < 0.05 vs. male ENU2-values [Student's *t*-test; *t*_(5)_ = 4.75].

To examine whether changes in the expression of mGlu5 receptors were gender-dependent, we performed a different set of experiments comparing female ENU2 mice with female heterozygous mice, and then male ENU2 mice with female ENU2 mice (no WT mice were available for these experiments). The analysis was restricted to the hippocampus. Expression of mGlu5 receptors did not differ between female ENU2 and heterozygous mice (Figure 3A). In addition, mGlu5 receptors had a significantly higher density in male ENU2 mice as compared to female ENU2 mice (Figure 3B).

### Down-regulation of the long isoforms of homer protein in the hippocampus and corpus striatum of ENU2 mice

**Figure 4:**
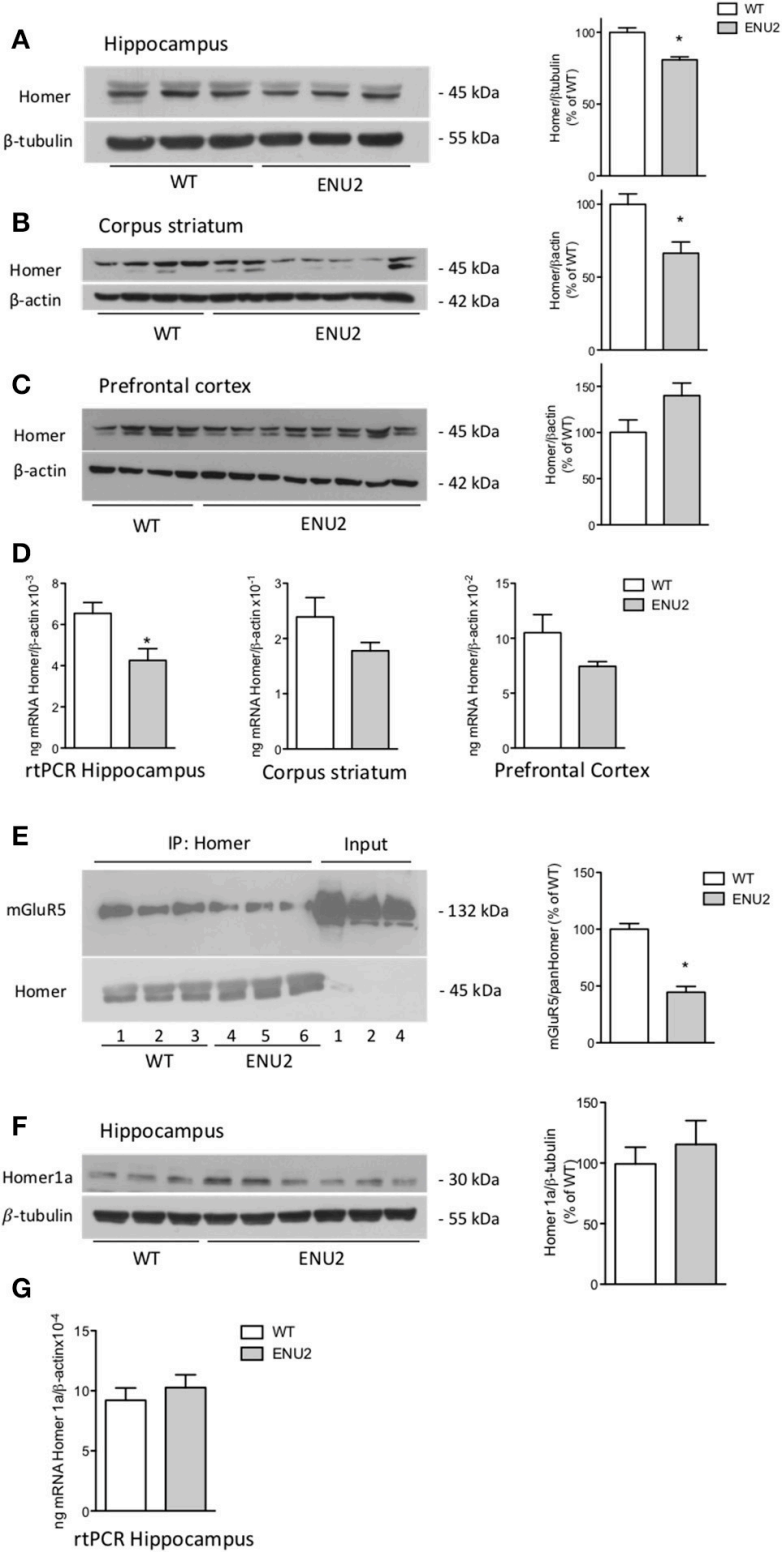
Reduced expression of the long Homer isoforms and reduced coupling of mGlu5 receptors to Homer in ENU2 mice. Representative immunoblots of long Homer isoforms in the hippocampus, corpus striatum, and prefrontal cortex of WT and ENU2 mice are shown in **(A)**, **(B)**, and **(C)**. Densitometric values are means ± S.E.M. of 6 WT and 9 ENU2 mice in **(A)**; 10 WT and 14 ENU2 mice in **(B)**; and 9 WT and 17 ENU2 mice in **(C)**. ^*^p < 0.05 vs. the respective WT-values [Student's *t*-test; *t*_(13)_ = 5.27 in **(A)**; *t*_(22)_ = 3.06 in **(B)**; *t*_(24)_ = 1.87 in **(C)**]. Pan-Homer 1 mRNA levels in the three brain regions of WT and ENU2 mice are shown in **(D)**. Values are means ± S.E.M. of 5 WT and 5 ENU2 mice for the hippocampus; 5 WT and 4 ENU2 mice for the corpus striatum; and 4 WT and 4 ENU2 mice for the prefrontal cortex. ^*^p < 0.05 vs. the respective WT-values [Student's *t*-test; *t*_(8)_ = 2.95 for the hippocampus; *t*_(7)_ = 1.47 for the corpus striatum; and *t*_(6)_ = 1.80, for the prefrontal cortex]. mGlu5 receptor protein levels in pan-Homer immunoprecipitates from the hippocampus of WT and ENU2 mice are shown in **(E)**. Densitometric values are means ± S.E.M. of 3 WT and 3 ENU2 mice. ^*^p < 0.05 vs. WT mice [Student's *t*-test; *t*_(4)_ = 7.74]. A representative immunoblot of the short Homer 1a isoform in the hippocampus of WT and ENU2 mice is shown in **(F)**. Densitometric values are means ± S.E.M. of 8 WT and 9 ENU2 mice. mRNA levels of Homer 1a in the hippocampus of WT and ENU2 mice are shown in **(G)**. Values are means ± S.E.M. of 5 WT and 6 ENU2 mice.

For Western blot analysis, we used a monoclonal antibody that detects all long Homer isoforms (Homer 1b,−1c,−2a–c, and −3). Immunoblots showed two major bands at about 45 kDa, and an additional faint band, which was exclusively visible in the hippocampus (Figures 4A–C). Long Homer isoform protein levels were significantly reduced in the hippocampus and corpus striatum of ENU2 mice as compared to their WT counterparts. The transcript of Homer 1 was also significantly reduced in the hippocampus of ENU2 mice, whereas a trend to a reduction was seen in the striatum and prefrontal cortex (Figures 4D). mGlu5 receptor protein levels were significantly reduced in pan-Homer immunoprecipitates from the hippocampus of ENU2 mice as compared to the hippocampus of WT mice (Figure 4E).

We also examined the expression of the inducible, short isoform of Homer (Homer 1a). Western blot analysis showed a single band at the expected molecular size (30 kDa). As opposed to the long isoforms of Homer, no changes in Homer 1a mRNA and protein levels were found in the hippocampus of ENU2 mice (Figures 4F,G).

### Analysis of mGlu5 receptor-dependent synaptic plasticity in ENU2 and WT mice

**Figure 5:**
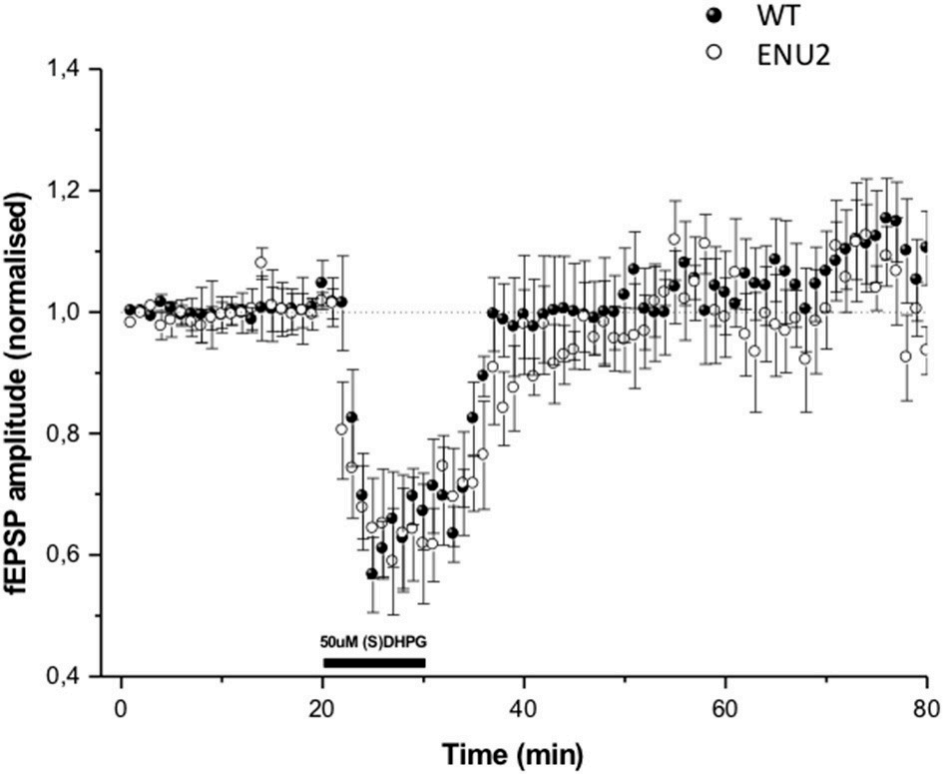
DHPG-induced changes in excitatory synaptic responses in the Schaeffer collateral/CA1 synapses of WT and ENU2 mice. No LTD could be induced in hippocampal slices prepared from the two genotypes. Values are means ± S.E.M. of data obtained from slices of 5–6 animals per group.

To examine whether changes in mGlu5 receptors and Homer proteins seen in the hippocampus of ENU2 mice could have an impact on mechanisms of activity-dependent synaptic plasticity, we measured responses to DHPG at the Schaffer collateral-CA1 synapses. In both ENU2 and WT mice DHPG (50 μM) induced a short-term depression (STD) of excitatory synaptic transmission, which returned back to normal 10 min after the termination of DHPG exposure. DHPG-induced STD did not differ between ENU2 and WT mice (Figure 5). We were unable to induce LTD in hippocampal slices of both genotypes, in line with the finding that mechanisms of synaptic plasticity are abnormal in the hippocampus of BTBR mice (MacPherson et al., 2008; Stephenson et al., 2011; Seese et al., 2014). This precluded any comparative study between ENU2 and WT mice on long-term forms of mGlu5 receptor-dependent synaptic plasticity.

### Pharmacological blockade of mGlu5 receptors improved behavioral abnormalities in ENU2 mice

**Figure 6:**
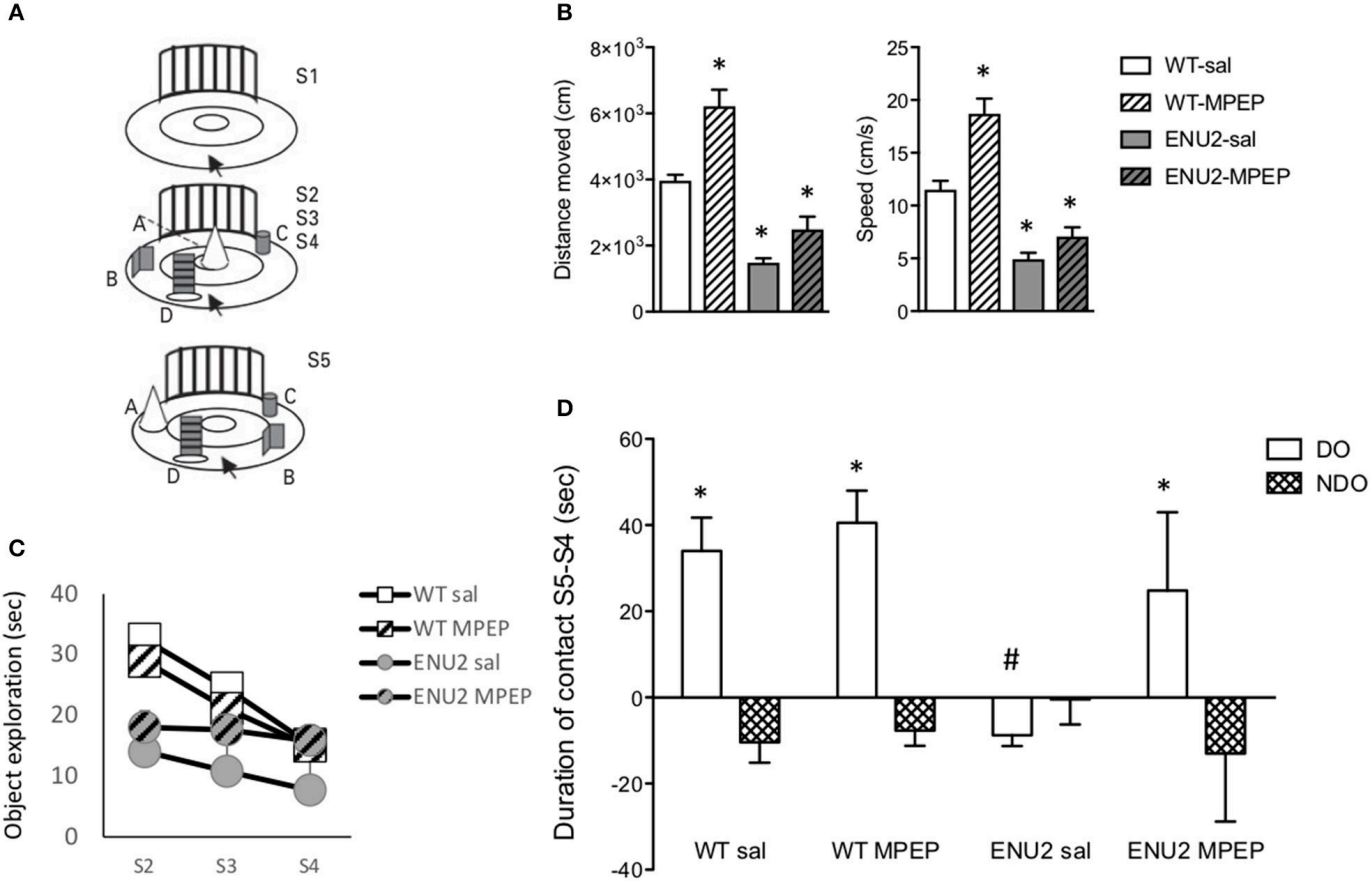
Pharmacological blockade of mGlu5 receptors improves cognitive performances in the spatial novelty test in ENU2 mice. The same open field arena **(A)** was used for the sequential assessment of locomotor activity (S1), object exploration (S2–S4), and spatial novelty (S5) in WT and ENU2 mice treated i.p. with either saline or MPEP (20 mg/kg). For the whole behavioral analysis we used 12 WT mice treated with saline, 7 WT mice treated with MPEP, 5 ENU2 mice treated with saline, and 5 ENU2 mice treated with MPEP. Values are always expressed as means ± S.E.M. Locomotor activity is expressed as distance moved and speed in **(B,C)**, respectively, where ^*^p < 0.05 vs. the respective values obtained in WT mice treated with saline (Two-way ANOVA + Fisher's LSD); genotype x treatment: **(B)** [*F*(_1, 24)_ = 6.15; *p* = 0.02; **(C)** *F*_(1, 25)_ = 4.95; *p* = 0.03]. Statistical analysis in **(D)** was performed by Three-way ANOVA + Fisher's LSD. Test × treatment: [*F*_(1, 32)_ = 4.37; *p* = 0.04]. *Post-hoc*: *p* < 0.05 vs. the respective NDO-values (^*^) or vs. all other DO-values (#). DO, Displaced Objects; NDO, Non Displaced Objects.

The same apparatus was used for measurements of locomotor activity (during the exploration phase) and for the analysis of how animals encode spatial relationships (spatial novelty test) (Figure 6A). In the exploratory phase (S1 in Figure 6A), ENU2 mice showed a large reduction in distance traveled and speed of movements as compared to WT mice. WT and ENU2 mice were treated i.p. with either vehicle or 20 mg/kg MPEP 30 min prior to the beginning of the behavioral sessions. All behavioral sessions (from S1 to S5) lasted for 42 min; thus, the time elapsed from the injection of MPEP (or vehicle) and the end of the behavioral sessions was 72 min. *In vivo* studies have shown that MPEP has a 75% receptor occupancy at 30 min and approximately 50% at 60 min following i.p. injection in C57Bl/6 mice. Receptor occupancy was higher in SD rats (Anderson et al., 2003). No studies on mGlu5 receptor occupancy were ever performed in BTBR mice. We used a 30-min pre-incubation time with MPEP on the basis of a previous report examining behavioral responses to MPEP in BTBR mice (Silverman et al., 2010). Acute systemic treatment with MPEP (20 mg/kg, i.p.) 30 min prior to the test session significantly enhanced locomotor activity in WT mice, whereas only a trend to an increase was observed in ENU2 mice (Figure 6B). In the first of the three sequential phases of object exploration (S2), ENU2 mice spent less time in object exploration as compared to WT mice. Treatment with MPEP had no effect on both ENU2 and WT mice in the S2 phase, corresponding to the acquisition phase of memory (Figure 6C). In the two subsequent phases (S3 and S4), corresponding to the consolidation phases of memory, WT mice treated with saline or MPEP, spent progressively less time in object exploration, as expected. A similar trend was observed with ENU2 mice treated with saline, but not with ENU2 mice treated with MPEP (Figure 6C). In the last phase of the test (S5) WT mice treated with saline or MPEP spent much more time than in S4 in exploring the two objects (objects labeled as “A” and “B” in Figure 6A) that had been displaced from their initial location (“displaced objects” or “DO” in Figure 6D). ENU2 mice spent less time than in S4 in exploring displaced objects. Interestingly, MPEP treatment dramatically enhanced the exploration time of displaced objects in ENU2 mice (Figure 6D). MPEP treatment had no effect on the exploration of non-displaced objects in both WT and ENU2 mice (Figure 6D).

## Discussion

Group-I mGlu receptors have been the focus of extensive investigation in animal models of ID and autism since Mark Bear, Kimberly Huber, and their Associates have shown that mGlu5 receptor-dependent LTD is amplified in the hippocampus of *Fmr1* knockout mice modeling Fragile-X syndrome. Changes in mGlu5 receptor signaling have been reported in animal models of autism spectrum disorders, such as Fragile-X, Rett's syndrome, Angelmann's syndrome, tuberous sclerosis, and chromosome 16p11.2 microdeletion (see Introduction and References therein).

The evidence that mGlu5 receptor protein levels were largely increased in the hippocampus and striatum of male ENU2 mice modeling PKU strengthens the relationship between mGlu5 receptors and the pathological phenotype of genetic disorders associated with ID. This increase likely reflects an enhanced stability or a reduced turnover rate of the mGlu5 receptor protein because no changes were found in the transcript of mGlu5 receptors in any of the selected brain region. An enhanced expression of mGlu5 receptors has also been found in *Fmr1* knockout mice, as well as in brain tissues from children affected by autism spectrum disorder. For example, an increased density of ^3^H-MPEP binding sites has been reported in the striatum of *Fmr1* knockout mice (Maccarrone et al., 2010), and an increase in mGlu5 receptor expression has been found in the prefrontal cortex of patients affected by Fragile-X syndrome (Lohith et al., 2013), in the brain of children with autism (Fatemi et al., 2011), and in brain specimens of patients affected by tuberous sclerosis (Boer et al., 2008).

mGlu5 receptors interact with Homer proteins, which critically regulate receptor expression, activity, and interaction with signaling proteins. Homer proteins bind to the C-terminus domain of mGlu5 receptors via their N-terminus EVH1 domain. Long isoforms of Homer, i.e., Homer 1b,−1c,−2, and −3, interact to each other through their C-terminal coiled-coil domains, and scaffolds mGlu5 receptors to effector proteins, such as the inositol-1,4,5-trisphosphate receptors, the ryanodine receptor, Shank, C-type transient receptor potentials, the phosphatidylinositol-3-kinase enhancer, and diacylglycerol lipase-alpha (DGLα). Homer 1a, a short Homer isoform lacking the coiled-coil domain, competes with long Homers in the binding to mGlu5 receptors and disrupts receptor interaction with effector proteins (Tu et al., 1998, 1999; Kammermeier et al., 2000; Feng et al., 2002; Gray et al., 2003; Rong et al., 2003; Yuan et al., 2003; Kim et al., 2006; Jung et al., 2007; Shiraishi-Yamaguchi and Furuichi, 2007; Worley et al., 2007). Conditions that tip the balance between long Homer isoforms and Homer 1a in favor of Homer 1a enhance the ligand-independent, constitutive activity of mGlu5 receptors (Ango et al., 2001).

We found a reduced expression of the long isoforms of Homer in the hippocampus and striatum of male ENU2 mice, whereas expression of Homer 1a was unchanged, at least in the hippocampus. mGlu5 receptor protein levels were reduced in pan-Homer immunoprecipitates from ENU2 mice, suggesting that mGlu5 receptors are less associated with the long isoforms of Homer protein, and, therefore might display a greater constitutive activity. A reduced association of mGlu5 receptors with long Homer isoforms has been reported in mouse models of monogenic autism, such as *Fmr1* and *Shank3* knockout mice (Giuffrida et al., 2005; Ronesi et al., 2012; Wang et al., 2016). Mice harboring a *Grm5* mutation that disrupts receptor interaction with Homer mimic several phenotypes of *Fmr1* knockout mice (Guo et al., 2016), and genetic deletion of Homer 1a, which results into an enhanced mGlu5 interaction with long Homer isoforms, corrects molecular and behavioral phenotype of *Fmr1* knockout mice (Ronesi et al., 2012). An opposite scenario was observed in Ube3A hemizygous mice modeling Angelman syndrome (another disorder characterized by autism and ID), in which Homer 1a levels are reduced in the hippocampus, resulting into an enhanced coupling of mGlu5 receptors with long Homer isoforms (Pignatelli et al., 2014). Thus, our findings obtained in ENU2 mice are in line with the involvement of the mGlu5/Homer axis in the pathophysiology of autism and ID.

Pharmacological blockade of mGlu5 receptors with selective NAMs, such as MPEP and CTEP, has been shown to correct several pathological phenotypes in mouse models of monogenic autism, such as *Fmr1* knockout mice (Michalon et al., 2012; Gandhi et al., 2014), BTBR T+tf/J mice (Silverman et al., 2010; Seese et al., 2014; Yang et al., 2015), mice exposed prenatally to valproic acid (Mehta et al., 2011), mutant mice modeling human chromosome 16p11.2 microdeletion (Tian et al., 2015), and *MecP2* knockout mice modeling Rett syndrome (Tao et al., 2016). *Tsc2*^−/−^ mice mimicking tuberous sclerosis are an exception because in these animals it is the activation of mGlu5 receptors that corrects the pathological phenotype (Auerbach et al., 2011).

The increased mGlu5 receptor protein levels combined with a reduced expression of long Homer isoforms in the hippocampus and striatum of ENU2 mice led us to hypothesize that an overactivity of mGlu5 receptors might contribute to the pathophysiology of cognitive dysfunction associated with PKU. We could not test whether mGlu5 receptor-dependent synaptic plasticity was abnormal in ENU2 mice because the mouse strain (BTBR) precluded the study of mGlu5-dependent LTD. We therefore examined whether behavioral abnormalities of ENU2 mice could be corrected by mGlu5 receptor blockade using MPEP. Systemic administration of MPEP in rodents is known to produce anxiolytic (Spooren et al., 2000; Tatarczynska et al., 2001; Pilc et al., 2002; Nordquist et al., 2007) and anti-Parkinsonian effects in rodents (Breysse et al., 2002; Coccurello et al., 2004; Levandis et al., 2008; De Leonibus et al., 2009) at doses that do not cause sedation. We tested the effect of MPEP on ambulation, object exploration, and spatial novelty discrimination in the same behavioral session. Untreated ENU2 mice showed a substantial reduction in locomotor activity, which may result from the reduced catecholamine and serotonin synthesis (Puglisi-Allegra et al., 2000), but can also be explained with the increased expression of mGlu5 receptors in the striatum. Activation of mGlu5 receptors on striatal projection neurons of the indirect pathway of the basal ganglia motor circuit restrains the action of dopamine at D2 receptors, thereby reducing motor activity (reviewed by Conn et al., 2005). Treatment with MPEP increased locomotor activity in ENU2 mice, but this effect was not specific because it was also observed in wild-type mice, as expected (Silverman et al., 2010). Where treatment with MPEP produced striking and genotype-specific effects was on the spatial novelty test, in which ENU2 mice showed a marked behavioral impairment reflected by the strong reduction in the time spent in exploring objects that had been displaced from their original position. MPEP treatment largely improved the performance of ENU2 mice in the spatial novelty test, without affecting behavior in wild-type mice. The spatial novelty test is a non-associative test that was designed to evaluate the ability of rodents to encode spatial relationships (Poucet, 1989; Roullet et al., 1997; Cabib et al., 2003). The hippocampus plays a crucial role in spatial information processing and novelty detection (Lisman and Otmakhova, 2001; Vinogradova, 2001; Lee et al., 2005; Hunsaker et al., 2008). Animal studies have shown that the hippocampus encodes information relative to the spatial aspects of object recognition, whereas the perirhinal cortex is involved in the processing of non-spatial aspects of object recognition (Brown and Aggleton, 2001; Aggleton and Brown, 2005). Thus, the spatial novelty test examines a form of hippocampus-dependent learning (Goh and Manahan-Vaughan, 2013), and the “therapeutic” effect of MPEP suggests that an overexpression/activity of mGlu5 receptors in the hippocampus contributes to the pathophysiology of cognitive dysfunction in ENU2 mice.

In conclusion, our findings provide a further example of a genetic disorder characterized by ID in which abnormalities of mGlu5 receptors can be detected, and suggest that pharmacological blockade of mGlu5 receptors might represent a novel strategy for the treatment of cognitive dysfunction in male PKU patients in whom the clinical outcome is suboptimal in spite of an early treatment with a Phe-deficient diet.

## Author contributions

FNa performed biochemical experiments and statistical analysis, wrote the first draft of the manuscript, approved the final version of the manuscript, and agrees to be accountable for all aspects of the work. RO and LI performed biochemical experiments, revised critically the manuscript, approved the final version of the manuscript, and agree to be accountable for all aspects of the work. LD and GB performed biochemical experiments during the revision phase, revised critically the manuscript, approved the final version of the manuscript, and agree to be accountable for all aspects of the work. MC and EF performed behavioral experiments, revised critically the manuscript, approved the final version of the manuscript, and agree to be accountable for all aspects of the work. SP and RN examined synaptic plasticity in hippocampal slices, revised critically the manuscript, approved the final version of the manuscript, and agree to be accountable for all aspects of the work. SP-A, FNi, VL, and TP designed experiments, revised critically the manuscript, approved the final version of the manuscript, and agree to be accountable for all aspects of the work.

### Conflict of interest statement

The authors declare that the research was conducted in the absence of any commercial or financial relationships that could be construed as a potential conflict of interest.
